# Seasonal Succession of Fungi Associated with *Ips typographus* Beetles and Their Phoretic Mites in an Outbreak Region of Finland

**DOI:** 10.1371/journal.pone.0155622

**Published:** 2016-05-17

**Authors:** Riikka Linnakoski, Saila Mahilainen, Alison Harrington, Henri Vanhanen, Miikka Eriksson, Lauri Mehtätalo, Ari Pappinen, Michael J. Wingfield

**Affiliations:** 1 Department of Forest Sciences, Faculty of Agriculture and Forestry, University of Helsinki, Helsinki, Finland; 2 Department of Microbiology and Plant Pathology, Forestry and Agricultural Biotechnology Institute (FABI), University of Pretoria, Pretoria, South Africa; 3 School of Forest Sciences, Faculty of Science and Forestry, University of Eastern Finland, Joensuu, Finland; 4 Watson Foundation, New York, United States of America; 5 Natural Resources Institute Finland (Luke), Joensuu, Finland; 6 School of Applied Educational Science and Teacher Education, Philosophical Faculty, University of Eastern Finland, Savonlinna, Finland; 7 School of Computing, University of Eastern Finland, Joensuu, Finland; Swedish University of Agricultural Sciences, SWEDEN

## Abstract

The ophiostomatoid fungi (*Microascales* and *Ophiostomatales*, Ascomycota) are common associates of *Ips typographus*, and include tree pathogens and species responsible for blue-stain of timber. Fungal assemblages associated with *I*. *typographus* have varied considerably between studies but few investigations have attempted to explain this variation. For this reason, we assessed the overall cultivable fungal diversity associated with *I*. *typographus* in a storm-felled spruce forest in south-eastern Finland. Fungi were isolated from the individually collected beetles as well as their phoretic mites in spring, summer and autumn, including different life stages of the beetle (hibernation, dispersal flight and first generation). The internal transcribed spacer (ITS) gene region was used to identify the fungi. A total of 32 operational taxonomic units (OTUs) were found and these resided in four fungal phyla/subphyla (24 Ascomycota, 2 Basidiomycota, 5 Mucoromycotina, 1 Mortierellomycotina) in association with adult bark beetles. Ophiostomatoid species were the most commonly detected fungal associates. A generalized linear model analysis showed a clear association between fungal communities and season, indicating seasonal succession among *I*. *typographus*-associated fungi. The season of sampling appears to be an important factor that has resulted in inconsistencies between results in previous studies. Many of these fungi were also found on phoretic mites and their presence or absence could have influenced variation in patterns of association.

## Introduction

The European spruce bark beetle (*Ips typographus*) is the most economically and ecologically important bark beetle species in coniferous forests of Europe. It infests mainly wind-felled and weakened trees and is capable of killing healthy trees in large numbers only when the population exceeds certain dangerous levels. *Ips typographus* is one of the driving forces of forest succession and, therefore, an important component of natural forest ecosystems [1]. However, mass outbreaks of the pest resulting in serious losses to forestry have more frequently been reported in Northern Europe. It has been suggested that climate change might alter both the frequency and the intensity of forest pest outbreaks [2–4]. Severity of *I*. *typographus* outbreaks has been linked to dry summers combined with warmer temperatures [4]. In this regard, climate change followed by increasing temperatures during the breeding season could directly affect the population dynamics of *I*. *typographus*, allowing the bark beetle to complete two rather than the normal one generation per year in Northern Europe [5]. Increased numbers of storms and drought periods associated with climate change will also indirectly increase the risk of bark beetle outbreaks via changes in availability of suitable breeding material [6].

During outbreaks, *I*. *typographus* colonizes trees via pheromone-mediated mass attacks and in concert with its fungal associates. This symbiosis has been hypothesized to exhaust host tree defenses [7–8]. However, bark beetles also vector fungi in non-outbreak conditions, and the positive role of the fungal associates in the tree-killing by bark beetles has been challenged [9]. Numerous studies have been conducted on the fungi associated with *I*. *typographus*, and particularly the biodiversity of ophiostomatoid [10] fungi belonging to *Microascales* and *Ophiostomatales* (Ascomycota) in Northern Europe (e.g. [11–23]) and *Geosmithia* fungi in Central and Northeastern Europe [24–25]. While fungal assemblages associated with this bark beetle species have varied considerably between studies, only a few investigations have attempted to explain this variation [17–18, 26–27].

The aim of the present study was to consider the seasonal variation in fungal assemblages associated with different stages of *I*. *typographus* in Finland. The overall fungal diversity associated with *I*. *typographus* including its phoretic mites in a storm-felled spruce forest in south-eastern Finland was investigated.

## Materials and Methods

### Study area and collection of bark beetles and mites

In July and August 2010, over 8 million m^3^ of tree timber was damaged in southern and middle Finland thunderstorms. Our study area was located in one of the most severely damaged forests in Ruokolahti, south-eastern Finland (61° 492’ N, 29° 054’ E). The surrounding stand is mainly *Myrtillus* type [28] with mature conifers dominated by *Picea abies*, and partly by *Pinus sylvestris*. The storm-felled trees comprised old-growth logs and there was a high volume of decaying wood present in the region. For these reasons salvage logging and removal of the storm-felled trees was difficult to conduct. The fallen trees were consequently left in the forest and a private 74 hectare forest preserve was established in 2010 in the most severely damaged region in Viitalampi, Ruokolahti. It was obvious that large volumes of suitable breeding material would result in an *I*. *typographus* outbreak in the region over the following years, especially where favorable weather conditions prevailed. The *I*. *typographus* population was monitored using pheromone trap catches and an assessment of beetle-killed trees. Spread of the bark beetle outbreak was controlled by sanitation logging in the surrounding areas. Bark beetles were collected adjacent to the newly established nature reserve area during 2013 for which the Tornator Oyj issued a research permit.

In April 2013, overwintering *I*. *typographus* adults were collected from the forest litter and under the bark of storm-felled trees. Each beetle hibernating under the bark was collected from a separate gallery. Samples of forest litter were collected approximately 0.5–1.0 m distance from the tree base, and 1–24 beetles were found from each litter sample. To avoid possible cross-contamination of the samples, all the bark beetles were collected individually with sterile forceps. Only living beetles were processed and each beetle was individually placed into a sterile 1.5 ml Eppendorf tube. During the dispersal flight period in early June 2013, bark beetles were lured to the outer surface of the trunks of the trap trees by using Ipsowit^®^ Standard (Witasek, PflanzenSchutz GmbH, Austria) pheromone strips. The first generation adults were collected at the end of October and early November from under the bark of storm-felled trees as well as newly attacked standing trees, and from the forest litter.

The beetles were transported to the laboratory and stored at -20°C for 24 hours. A bark beetle species morphologically similar to *I*. *typographus*, *Ips amitinus*, was also present in the studied region. Therefore, after freeze-treatment, the identification of each beetle was confirmed under dissection microscope and *I*. *amitinus* individuals were excluded from this study. At the same time, all phoretic mites present on the beetles were collected individually and transferred to new sterile Eppendorf tubes. We did not attempt to identify the mites. The tubes containing beetles and mites were stored at 5°C and fungal isolations were made from them on the same day that they were identified.

### Isolation and morphological grouping of fungi

Each bark beetle and phoretic mite was individually crushed and placed onto the surface of Malt Extract Agar (MEA; 2% malt extract from Biokar Diagnostics, Beauvais, France and 2% agar from Fisher Scientific, Mexico) in Petri dishes containing 0.05 g/l of streptomycin sulphate (Sigma-Aldrich, China). The plates were then incubated at 25°C for 2–4 weeks and observed daily for fungal growth. When fungal growth was observed, spore masses and/or fungal mycelia were transferred to fresh 2% MEA plates (without antibiotics) and sub-cultured until pure cultures were obtained. Purified fungal isolates were then examined under dissection microscope and grouped based on their colony characteristics. Depending on the size of the morphological group, 1–4 isolates from each group were chosen for identification based on DNA sequence comparisons. Representative isolates of ophiostomatoid fungi were deposited in the culture collection (CMW) of the Forestry and Agricultural Biotechnology Institute (FABI), University of Pretoria, South Africa.

### DNA extraction, amplification and sequencing

Purified fungal isolates were grown on 2% MEA in 7 cm Petri dishes at 25°C for up to 2 weeks prior to DNA extraction. Genomic DNA was extracted using PrepMan^™^ Ultra Sample preparation reagent (Applied Biosystems, Foster City, CA, USA). The molecular marker used in this study was the internal transcribed spacer (ITS) region of the nuclear ribosomal DNA, which has its limitations but is generally sufficient for most fungi at least for the reliable identification at the species complex level. The ITS gene region was amplified using a primer pair ITS1-F [29] and ITS4 [30].

Amplification of the studied gene region and purification of the PCR products were performed using the same protocols described in our previous publication [31]. The PCR reaction mixture contained 0.15 μL of MyTaq^™^ DNA Polymerase (5 U/μl) (Bioline, Massachusetts, USA), 2.5 μl of MyTaq^™^ Reaction Buffer (5×) containing dNTPs, MgCl_2_ and enhancers for the optimal performance, and 0.50 μl of each primer (Whitehead Scientific Ltd, Cape Town, South Africa). PCR reactions were performed using an ABI 2720 Thermal Cycler (Applied Biosystems, Foster City, CA, USA) as follows: an initial denaturation step at 95°C for 2 min, followed by 35 cycles of 30 s at 94°C, 30 s at 55°C and 1 min at 72°C, and a final chain elongation at 72°C for 7 min. PCR products were visualized under UV light after staining 5 μl aliquots with 2 μl of GelRed^™^ Nucleic Acid Gel Stain (Biotium, Hayward, CA, USA) and separation on a 1% agarose gel. Successfully amplified products were purified using the Exo-SAP protocol: the remaining PCR product (20 μl) was mixed with 8 μl of Exo-SAP [5 μl of Exonuclease I (20 U/μl) (Fermentas,Vilnius, Lithuania) and 100 μl of Shrimp Alkaline Phosphatase (1 U/μL) (Roche Diagnostics, Indianapolis, USA) in a 1000 μl reaction mixture] and incubated at 37°C for 15 minutes and following immediate incubation at 80°C for 15 minutes.

The cycle sequencing reactions were performed in a 12 μl reaction mixture. The reaction mixture contained 0.5 μl of BigDye^®^ Terminator v3.1 Ready Reaction mixture (Perkin-Elmer Applied Biosystems, Warrington, UK), 2.1 μl of sequencing buffer, 1 μl of either the forward or reverse primer (10 mM) and 2 μl of cleaned PCR product. Sequencing was done in both directions using same primers as used for amplification. The thermal cycling conditions for the sequencing reactions were: 25 cycles of 10 s at 96°C, 5 s at 55°C and 4 min at 60°C. The sequencing products were cleaned using ethanol/salt precipitation and dried in a laminar flow bench overnight. Sequencing was done on an ABI Prism 3100 DNA Analyzer (Applied Biosystems, Foster City, CA, USA) located at the DNA Sequencing Facility of the Forestry and Agricultural Biotechnology Institute (FABI), University of Pretoria.

### DNA sequence analyses and fungal identification

Geneious R6 software (Biomatters Ltd, Auckland, New Zealand) was used to assess the quality of sequence chromatograms, to edit (when necessary) and trim the 5′ and 3′ends to uniform length, and to compile the consensus sequences. Fungal identification to the species level was made as far as possible. This was estimated individually for each isolate with caution given to the fact that the ITS sequence variability in certain fungal groups is low, and acknowledging possibly misidentified sequences in GenBank. Isolates were identified using a megablast algorithm implemented in GenBank nucleotide database (http://blast.ncbi.nlm.nih.gov/Blast.cgi). We considered reliable identification to consist of the BLAST matches that had ≥98% sequence similarity to ex-type sequences or peer-reviewed published studies. The sequences of isolates that represented the same species based on the BLAST search were compiled in the same data set using Molecular Evolutionary Genetic Analysis (MEGA) v. 6 [32]. The data sets were aligned using MAFFT v. 7 online version [33] with the FFT-NS-i strategy with a 200PAM/κ = 2 scoring matrix, a gap opening penalty of 1.53 and an offset value of 0.00. The sequence similarities were then visualized and compared in MEGA v. 6 and the sequences with a ≥98% similarity were assigned to the same species.

### Statistical analyses

The number of fungal species per beetle was analysed using Poisson GLM with log link. The model is specified as *Y*_*i*_~*Poisson*(*μ*_*i*_), where *Y*_*i*_ is the number of fungal species in bark beetle *i*, and

$$\mu_{i}=\text{exp}\left(\beta_{0}+\beta_{1}S_{i}+\beta_{2}A_{i}+\beta_{2}m_{i}\right)$$

where *S*_*i*_ and *A*_*i*_ are binary predictors indicating whether bark beetle *i* was collected in summer and autumn respectively, and *m*_*i*_ indicates the centralized number of mites found in bark beetle *i* (i.e. observed number of mites—mean number of mites in the data); *β*_0_, *β*_1_, *β*_2_, and *β*_3_ are the regression coefficients associated with the predictors. The same model was fitted both for the total number of fungal species and for the total number of ophiostomatoid species per bark beetle.

Poisson GLM is a theoretically justified model for independent counts that do not have an upper limit [34]. The parameter *μ*_*i*_ specifies both the mean and variance of the fungal count for beetle *i*. Lack of independence in the data would lead to over-dispersion (variance is larger than the mean) or under-dispersion (variance is lower than the mean), and ignoring existing over- or under-dispersion in modelling leads to problems in tests on the regression coefficients. In this study, potential over-dispersion could be caused by co-association between fungal species. Correspondingly, under-dispersion could imply that some fungi tend to exclude each other. The model was fitted using the method of Maximum Likelihood using R- function glm [35].

## Results

### Collection of bark beetles and mites

In total, 298 adult beetles were collected. During the spring, 97 beetles were collected, of which 44 were found from the soil litter and 53 under the bark. During the main swarming period in summer, 101 beetles were collected. In the autumn, 100 first generation beetles were collected, of which 54 were from the forest litter and 46 were found under the bark. Phoretic mites were found on 13.1% of adult beetles. The numbers of mites carried by these beetles was greatest in the autumn, when 22 mites were found, 6 of which were from the beetles in the litter and 16 were from the beetles under the bark. In the spring, 15 mites were found all from beetles in the litter. In the summer, only 2 mites were found from the dispersing beetles. When mites were present, the number per individual beetle ranged from 1 to 4, the mean number for all beetles being 0.12 mites per beetle.

### Isolation and identification of fungi

Fungal associates were isolated from 68.5% of the collected beetles and 94.1% of the mites. The study resulted in 402 fungal strains isolated from the beetles, and 52 strains isolated from the mites. Grouping of the isolates based on culture morphology resulted 104 morphological groups. In total 129 isolates were selected for DNA sequencing. Sequencing the ITS region failed in the case of eight morphological groups. The lengths of trimmed consensus sequences varied from 500 bp to 650 bp. The sequences obtained in this study were deposited in GenBank and their accession numbers are provided in the Table 1.

**Table 1:** Representative isolates of *I*. *typographus*-associated fungi collected in this study.

Fungal OTU	CMW no.^1^	GenBank acc. no.
**Ascomycota**		
*Alternaria arbusti*		KT896627
*Arthrinium* sp.		KT896628
*Beauveria pseudobassiana*		KT896629
*Bipolaris* sp.		KT896630
*Botrytis cinerea*		KT896631
*Cladosporium* sp.		KT896633
*Cosmospora vilior*		KT896634
*Endoconidiophora polonica*	43745	KT896632
*Graphium fimbriisporum*	43744	KT896635
*Grosmannia cucullata*	43737	KT896636
*G*. *olivaceae*	43741	KT896637
*G*. *penicillata*	43738	KT896638
*G*. *piceiperda*	43739	KT896639
*Grosmannia* sp.	43743	KT896640
*Monilinia* sp.		KT896641
*Ophiostoma ainoae*	43718	KT896642
*O*. *bicolor*	43723	KT896643
*O*. *brunneo-ciliatum*		KT896644
*O*. *piceae*	43732	KT896645
*O*. *tetropii*	43736	KT896649
*Phaeosphaeria vagans*		KT896646
*Phaeosphaeria* sp.		KT896647
*Phoma* sp.		KT896648
*Trichoderma polysporum*		KT896652
**Basidiomycota**		
*Schizophyllum commune*		KT896650
*Trametes* sp.		KT896651
**Mortierellomycotina**		
*Mortierella humilis*		KT896653
**Mucoromycotina**		
*Absidia* sp.		KT896654
*Basidiobolus magnus*		KT896655
*Mucor hiemalis f*. *hiemalis*		KT896656
*Umbelopsis isabellina*		KT896657
*Umbelopsis* sp.		KT896658

^1^ The culture collection (CMW) of the Forestry and Agricultural Biotechnology Institute (FABI), University of Pretoria, South Africa

Based on the molecular identification, fungi isolated in this study were assigned to 32 operational taxonomic units (OTUs) in four different fungal phyla: Ascomycota (24 species), Basidiomycota (2 species), Mortierellomycotina (1 species) and Mucoromycotina (5 species) (Table 1). The ophiostomatoid fungi were the most numerous fungi, and represented as 256 strains (63.7% of all fungi) isolated from the beetles, and 45 strains (86.5% of all fungi) isolated from the mites. Also other fungi (molds and yeasts) were frequently found but not recorded in this study.

Twelve ophiostomatoid species were detected. These included eleven species that were assigned to known species, including *Endoconidiophora polonica*, *Graphium fimbriisporum*, *Grosmannia cucullata*, *Grosmannia olivacea*, *Grosmannia penicillata*, *Grosmannia piceiperda*, *Ophiostoma ainoae*, *Ophiostoma bicolor*, *Ophiostoma brunneo-ciliatum*, *Ophiostoma piceae*, and *Ophiostoma tetropii* (Tables 1 and 2). One *Grosmannia* species could not be assigned to any currently known species, thus representing a putatively novel taxon.

**Table 2:** Seasonal proportions of *Ips typographus*-associated fungi isolated in this study.

	Proportion (%)					
Fungal OTU	Spring		Summer	Autumn		Total
	litter	bark		litter	bark	
**Ascomycota**						
*Alternaria arbusti*	0	0	2.13	0	0	1.24
*Arthrinium* sp.	0	0	0.85	0	0	0.50
*Beauveria pseudobassiana*	0	0	1.70	0	1.41	1.24
*Bipolaris* sp.	1.04	0	13.19	0	1.41	8.21
*Botrytis cinerea*	1.04	1.04	10.64	0	0	6.72
*Cladosporium* sp.	0	0	8.51	0	0	4.98
*Cosmospora vilior*	0	0	0	0	1.41	0.25
*Endoconidiophora polonica*	3.13	3.13	10.64	4.23	1.41	8.71
*Graphium fimbriisporum*	2.08	2.08	0.85	1.41	1.41	1.99
*Grosmannia cucullata*	0	0	0.43	0	1.41	0.50
*G*. *olivaceae*	0	0	0.43	0	0	0.25
*G*. *penicillata*	2.08	7.29	0.85	0	1.41	2.99
*G*. *piceiperda*	6.25	9.38	2.55	4.23	8.45	7.46
*Grosmannia* sp.	1.04	2.08	2.55	0	1.41	2.49
*Monilinia* sp.	0	0	2.13	0	0	1.24
*Ophiostoma ainoae*	4.17	4.17	12.77	2.82	0	9.20
*O*. *bicolor*	12.50	18.75	22.13	12.68	12.68	24.88
*O*. *brunneo-ciliatum*	0	0	0.43	0	0	0.25
*O*. *piceae*	2.08	2.08	1.70	1.41	0	2.24
*O*. *tetropii*	1.04	0	0.43	5.63	4.23	2.24
*Phaeosphaeria vagans*	0	0	1.28	0	0	0.75
*Phaeosphaeria* sp.	0	0	0.43	0	0	0.25
*Phoma* sp.	1.04	0	0.85	1.41	0	1.00
*Trichoderma polysporum*	0	0	0.43	0	0	0.25
**Basidiomycota**						
*Schizophyllum commune*	0	1.04	0	0	0	0.25
*Trametes* sp.	0	0	0.43	0	0	0.25
**Mortierellomycotina**						
*Mortierella humilis*	2.08	1.04	0.85	5.63	2.82	2.74
**Mucoromycotina**						
*Absidia* sp.	2.08	0	0	8.45	0	1.99
*Basidiobolus magnus*	0	0	0	1.41	0	0.25
*Mucor hiemalis f*. *hiemalis*	0	0	0.85	4.23	1.41	1.49
*Umbelopsis isabellina*	6.25	2.08	0	5.63	0	2.99
*Umbelopsis* sp.	1.04	0	0	0	0	0.25
***N* of beetles**	44	53	101	54	46	298
***N* of fungal isolates**	47	49	235	41	30	402
***N* of fungal species**	16	12	26	13	13	32
**Mean no of ophiostomatoid species per beetle**	1.50	1.33	1.73	1.38	1.71	1.58
**Beetles carrying at least one ophiostomatoid species (%)**	50.00	62.26	76.24	29.63	30.43	54.36

The ITS data did not provide sufficient resolution for ophiostomatoid species delineation within the cryptic species complexes [36]. Therefore, the fungus identified here as *O*. *brunneo-ciliatum* probably presents a cryptic novel species similar to *O*. *brunneo-ciliatum* (Linnakoski et al. unpublished). Overall, *O*. *bicolor*, *O*. *ainoae*, *E*. *polonica* and *G*. *piceiperda* were the most frequently isolated ophiostomatoid fungi associated with the adult beetles (Table 2). These same species were the most frequently found isolates associated with mites, except for *O*. *ainoae* that was never detected (Table 3).

**Table 3:** Seasonal proportions of fungi isolated from mites phoretic on *Ips typographus*.

	Proportion (%)					
Fungal OTU	Spring		Summer	Autumn		Total
	litter	bark		litter	bark	
**Ascomycota**						
*Cosmospora vilior*	0	0	0	0	2.63	1.92
*Endoconidiophora polonica*	16.67	0	0	5.26	0	7.69
*Graphium fimbriisporum*	8.33	0	0	0	2.63	3.85
*Grosmannia cucullata*	0	0	0	0	10.53	7.69
*G*. *penicillata*	0	0	0	0	10.53	7.69
*G*. *piceiperda*	0	0	50	0	18.42	15.38
*Grosmannia* sp.	0	0	50	0	2.63	3.85
*O*. *bicolor*	50.00	0	0	10.53	23.68	36.54
*O*. *tetropii*	0	0	0	0	10.53	7.69
**Mortierellomycotina**						
*Mortierella humilis*	8.33	0	0	0	0	1.92
**Mucoromycotina**						
*Absidia* sp.	0	0	0	2.63	0	1.92
*Umbelopsis isabellina*	16.67	0	0	0	0	3.85
***N* of mites**	15	0	2	6	16	39
***N* of fungal isolates**	12	0	2	7	31	52
***N* of fungal species**	5	0	2	3	8	12
**Mean no of ophiostomatoid species per mite**	0.9	0	2.0	1.67	1.5	1.40
**Mites carrying at least one ophiostomatoid species (%)**	75	0	50	83.33	93.75	76.92

The most numerous non-ophiostomatoid Ascomycota included *Bipolaris* sp., *Botrytis cinerea* and *Cladosporium* sp. that were present reasonably frequently (Table 2). Most non-ophiostomatoid species were detected only occasionally. The isolates belonging to Basidiomycota, Mortierellomycotina, and Mycoromycotina included species that were typically present only in low numbers in association with adult beetles or their phoretic mites (Tables 2 and 3).

There were differences between the fungal assesmblages from beetles collected from different overwintering environments. Adults of *I*. *typographus* that overwintered in the forest litter were exlusively or at least commonly associated with soil-born fungi (e.g. *Absidia*, *Mucor* and *Umbelopsis*) (Table 2). The majority of the ophiostomatoid species were detected from both overwintering substrates. Amongst the ophiostomatoid fungi, *E*. *polonica* was found more frequently from beetles and mites hibernating in the forest litter, and *Grosmannia* species were detected more commonly from beetles and their phoretic mites hibernating under the bark (Tables 2 and 3).

### Occurrence of fungi in different seasons

The fungal species richness and number of isolates detected from *I*. *typographus* were highest during the bark beetle dispersal flight, when in total 235 isolates assigned to 26 species were detected (Table 2). In addition, the percentage of beetles carrying ophiostomatoid species was highest in summer, when 76% of the beetles carried at least one ophiostomatoid species compared to autumn, when only 30% of the beetles were associated with ophiostomatoid fungi. *Ophiostoma bicolor* was the predominant species in all seasons. The other species most commonly detected included *E*. *polonica*, *G*. *piceiperda*, *G*. *penicillata* and *Umbelopsis isabellina* in the spring. During the bark beetle dispersal flight, *Bipolaris* sp., *O*. *ainoae*, *B*. *cinerea* and *E*. *polonica* were the most commonly detected species. *Ophiostoma tetropii* and most species belonging to Mortierellomycotina and Mycoromycotina were mainly detected in autumn.

The observations were supported by the generalized linear modeling that identified a clear association between fungal communities and season. The mean number of fungal species per bark beetle in spring was 0.99 species per beetle. It increased to 2.37 (2.404*0.985) species in summer and thereafter decreased to 0.700 (0.985*0.710) species per beetle in autumn (Table 4). The difference between summer and two other seasons was highly significant (p<0.000) and the difference between spring and autumn was significant (p = 0.029). The number of mites did not have statistically significant (p = 0.259) positive effect on the number of fungal species per beetle. Very similar results were obtained for ophiostomatoid fungi species, where the mean number of fungal species in spring, summer and autumn were 0.79, 1.33 and 0.45, respectively, and the differences between seasons were highly significant. The data did not show clear signs of over- or under-dispersion, therefore the data did not indicate co-existence or exclusion of fungal species.

**Table 4:** Parameter estimates (displayed against spring) for the Poisson GLM for all fungi (left) and for ophiostomatoid fungi.

	All fungi				Ophiostomatoid fungi			
	$\hat{\beta }$	$exp\;(\hat{\beta )}$	s.e.	p-value	$\hat{\beta }$	$exp\;(\hat{\beta )}$	s.e.	p-value
**Constant**	-0.015	0.985	0.102	0.883	-0.239	0.788	0.115	0.037
**Summer**	0.877	2.404	0.123	0.000	0.524	1.688	0.145	0.000
**Autumn**	-0.342	0.710	0.157	0.029	-0.559	0.572	0.187	0.003
**Mites**	0.120	1.128	0.106	0.259	0.157	1.170	0.118	0.184

## Discussion

Clear associations were found between fungal communities and season in this study and these coincide with the life stages of *I*. *typographus*. This suggests seasonal succession among *I*. *typographus* associated fungi. The data showed that *I*. *typographus* vectors a large diversity of fungi. Of these, a total of 32 operational taxonomic units (OTUs) resided in four fungal phyla/subphyla (24 Ascomycota, 2 Basidiomycota, 5 Mucoromycotina and 1 Mortierellomycotina) based on the fungal barcode (ITS) gene region. The study is also the first to provide preliminary information regarding the fungi associated with mites phoretic on *I*. *typographus* in Finland.

The fungal species richness and the number of fungal isolates varied between the seasons sampled. Some of the fungal species were detected only in a certain season, e.g. *Schizophyllum commune* in the spring, *Alternaria arbusti* in the summer, and *Cosmospora vilior* in the autumn. The majority of the ophiostomatoid fungi were present in all the seasons. Some of these fungi were detected more commonly in a specific season, e.g. *G*. *penicillata* in the spring, *E*. *conidiophora* in the summer, and *O*. *tetropii* in the autumn. The beetle’s overwintering site also affected the fungal associates of *I*. *typographus*. Interestingly, *Grosmannia* species were found more commonly on beetles and mites that hibernated under the host tree’s bark. This observation supports the results of a previous study where *Grosmannia* species were more often found on beetles collected under the bark [21]. Most of the fungi were detected during the dispersal flight period of *I*. *typographus*. The high number of different fungi during the dispersal flight is most probably due the fact that *I*. *typographus* can disperse over long distances [37–40]. Therefore, the individuals caught in this study would likely have originated from quite a large area. Interestingly, the individual fungal species occurred independently of each other. This is consistent with the results of a previous study where no correlation was found among different fungal species associated with *I*. *typographus* [41].

The fungal species assemblage associated with *I*. *typographus* in the present study was consistent with that found in previous studies conducted in Europe [11–12, 15, 19–22, 26–27, 41–49]. The fungal assemblage is known to consist mainly of ascomycetes, which were also the most common fungi in this study. Of these, the biodiversity of ophiostomatoid fungi belonging to orders *Microascales* and *Ophiostomatales* (Ascomycota) are best known in this niche [10, 50] and they were the most numerous species found in this study. The fungi detected in low numbers were most likely only incidental associates without particular relevance to the vector insect or its associated mites. These include wood-decaying basidiomycetes, which have been only rarely detected [11, 21, 27, 51]. The results are also consistent with those of previous studies [24–25] that describe associations between the bark beetles and *Geosmithia* spp. Lack of *Geosmithia* species in the present study confirmed that these fungi are rare components of the *I*. *typographus* mycobiome.

The diversity of ophiostomatoid species detected in this study was not surprising. The fungi most typically reported in association with *I*. *typographus* in Europe include *E*. *polonica*, *G*. *penicillata*, *G*. *piceiperda*, *O*. *ainoae* and *O*. *bicolor* [11–12, 15, 20–23, 26, 41–49, 52]. Other ophiostomatoid species including *Ceratocystiopsis minuta*, *G*. *cucullata*, *G*. *olivacea*, *O*. *flexuosum*, *O*. *piceae*, *O*. *tetropii* and *Graphium* spp. have also been reported in more than one study, but these species are generally considered rare associates of *I*. *typographus* [20, 43, 46].

It is relevant to consider that substantial quantitative differences have been found between different studies of the ophiostomatoid fungi associated with *I*. *typographus*, and not all species are consistently found in all studies. The results of the present study suggest that these differences are linked to sampling time. For example, our previous studies [22–23] considered the fungi from beetles constructing galleries during July-August in Finland and neighboring Russia failed to detect *G*. *penicillata*. This is in contrast to other studies where this fungus occurred commonly in Fennoscandia [11–12, 14–18, 42, 52]. The results of the present study suggest that *G*. *penicillata* favors the later stages of fungal succession in bark beetle galleries. It is, therefore, more likely to be found in association with the hibernating beetles than in fresh bark beetle galleries. This in agreement with other studies that suggesting that this fungus is a secondary colonist, better adapted infecting weakened and dead wood [11, 16].

Numerous studies have reported the occurrence of the pathogenic fungus *E*. *polonica* to be highly variable, see e.g. [12, 15, 19–23, 27, 41, 46–47, 53]. The role of this highly pathogenic fungus in the tree-killing by *I*. *typographus* has been the subject of discussion and debate [7–9, 54]. *Endoconidiophora polonica* was one of the most commonly encountered species in the present study. It was detected mainly during the bark beetle dispersal flight in summer, but was also regularly detected in the other seasons. The present study supports the view that *E*. *polonica* is an early colonist of spruce sapwood following the spruce bark beetle attack [11, 15–17, 26, 55].

Several other fungi commonly found in association with *I*. *typographus* are considered non-pathogenic or weakly aggressive [11, 20, 26, 43, 56–57]. One of these species, *O*. *bicolor*, was the predominant species encountered during all seasons in association with adult *I*. *typographus*, indicating a specific association with the vector beetle. This is consistent with previous investigations conducted in Europe (e.g. [15–20, 26–27, 41, 47–48, 53, 58]) as well as Japan [59]. *Ophiostoma bicolor* was also the predominant species found in association with mites in this study.

An increasing number of studies have been devoted to studying phoretic mites associated with bark beetles and their roles in fungal transmission [60–65]. Mite species associated with *I*. *typographus* in Finland have been unknown until a recent study [66]. The present study was based on a relatively small sample and provides only preliminary insight into fungal diversity associated with mites carried by *I*. *typographus* in Finland. We found phoretic mites on 13% of adult beetles similar to a study conducted in Bulgaria that reported 11% of *I*. *typographus* inviduals to carry mites [67]. But much greater numbers were reported in another study [61]. Mites were more numerous during spring and autumn, and almost absent during the dispersal flight. The presence of mites can significantly decrease the insect capacity to disperse [68], which could have influenced the results of this study. The common association with mites and *O*. *bicolor* has also been reported in other studies that have investigated *I*. *typographus* and *I*. *typographus japonicus* [60–61].

Several factors other than the season of sampling could influence the detection of bark beetle-associated fungi, thus obscuring the results and complicating comparisons between different studies. Factors that could influence fungal communities include geographical and climatic differences [69], forest microsite, and forest management history in the study site. Other factors include sampling and isolation methodology (e.g. type of samples, method of sample disinfection, and culture medium). Following sample collection, different techniques have been applied for fungal identification ranging from morphological to a variety of molecular methods. Particularly, the morphological characters of ophiostomatoid fungi that include several cryptic species are subjective and can provide obscure results. Detection of fungi in this study was based on culture-based methods. Previous studies have shown that culture-based methods accompanied with sequencing of clone libraries would provide complementary information on fungal assemblages [21, 27, 51, 70]. In addition, high-throughput sequencing methods provide new possibilities for studying fungal communities although these have not yet been tested with *I*. *typographus*.

The present study identified a clear association between fungal communities and season, with mean number of associated fungal species highest during the dispersal flight. The results also provide evidence for fungal species-specific differences in their seasonal occurrence in association with *I*. *typographus*. We, therefore, recommend that seasonal variation should be taken into account in future studies investigating overall bark beetle-associated fungal diversity and risk evaluation related to these interactions. Because *I*. *typographus* and its fungal associates pose global risks that are related to accidental introduction into new environments linked to global trade of untreated timber or as a result of a climate change, more extensive studies should be undertaken to gain a better understanding of seasonal and temporal differences among *I*. *typographus*-associated fungi. Furthermore, current knowledge regarding beetle-mite-fungus interactions is very limited and further studies are required to gain a better understanding of their ecological and potential economic importance.
